# Malaria elimination does not cost more than malaria control: Sri Lanka a case in point

**DOI:** 10.1186/s12936-022-04249-9

**Published:** 2022-08-01

**Authors:** Kamini Mendis, Rajitha Wickremasinghe, Risintha Premaratne

**Affiliations:** 1 Department of Parasitology, Faculty of Medicine, University of Colombo, 25 Kynsey Road, Colombo, Sri Lanka; 2 Department of Public Health, Faculty of Medicine, University of Kelaniya, P.O. Box 6, Thalagolla Road, Ragama, 11010 Sri Lanka; 3 Anti Malaria Campaign, 555/5 Public Health Building, Narahenpita, Sri Lanka; 4 Present Address: World Health Organization Regional Office for South East Asia, New Delhi, India

**Keywords:** Cost of malaria elimination, Cost of malaria control, Malaria elimination in Sri Lanka

## Abstract

**Background:**

Malaria was endemic in Sri Lanka for centuries and was eliminated in 2012. It is widely assumed that the costs of elimination are generally greater than that of control. The costs of malaria elimination in Sri Lanka with that of malaria control in the past using periods in which starting transmission dynamics were similar were compared.

**Methods:**

The expenditure of the Anti-Malaria Campaign (AMC), total and by budget category, during 2002–2010 is compared with that of malaria control during the period 1980–1989, using regression analyses and the Mann Whitney U statistic.

**Results:**

The expenditure on malaria control and malaria elimination was similar ranging from 21 to 45 million USD per year when adjusted for inflation. In both periods, external funding for the malaria progamme constituted around 24% of the total budget; during the control phase in the 1980s, external funds came from bilateral agencies and were disbursed in accordance with government budget guidelines. In the elimination phase in the 2000s, most of external funding was from the Global Fund and had flexibility of disbursement. In the 1980s, most funds were expended on commodities—insecticides, diagnostics and medicines and their delivery; in the elimination phase, they were spent on programme management, human resources, technical assistance and monitoring and evaluation; monitoring and evaluation was not a budget line in the 1980s. Although the cost per case of malaria was considerably higher during the elimination phase than in the control phase, expenditure was not on individual cases but on general systems strengthening.

**Conclusion:**

Malaria elimination in Southeast Asia may not require more funding than malaria control. But sustained funding for an agile programme with flexibility in fund utilization and improved efficiencies in programme management with stringent monitoring and evaluation appears to be critically important.

## Background

In 2012, malaria was eliminated from Sri Lanka, a tropical island with a population of 21 million, which was endemic for malaria for centuries. It received World Health Organization (WHO) certification as a malaria-free country in 2016 [1, 2]. In 1963, a failed attempt at elimination during the Global Malaria Eradication Programme reduced malaria to just 17 cases. However, the disease resurged soon after and persisted at endemic levels for 50 more years despite a malaria control programme being in operation throughout [3].

There is currently, a heightened global interest in eliminating malaria from countries [4], keeping in view a longer-term aim of malaria eradication [5–7]. Insights from countries that have successfully eliminated malaria may serve to inform both national and global policies that underpin the current global malaria elimination drive. Factors that may have been particularly important in determining the success of elimination from Sri Lanka have been previously identified [8]; this paper focuses on the financial costs of elimination, examining how it compared with the cost of malaria control in the past.

Several published reports on the costs incurred by malaria control and elimination programmes including several during the Global Malaria Elimination Programme have been subjected to a systematic review [9]. The review found that the investments needed for malaria control and elimination varied greatly between countries and contexts, and that the costs of elimination were, in most, greater than that of control. That, conclusion however, was based on secondary data from different countries and contexts at different periods of times. The work reported here constitutes the first using primary data on investments in malaria control and elimination from the same country during periods of comparable malaria transmission patterns and may provide fresh insights into the cost-benefit argument for malaria elimination.

**Fig. 1:**
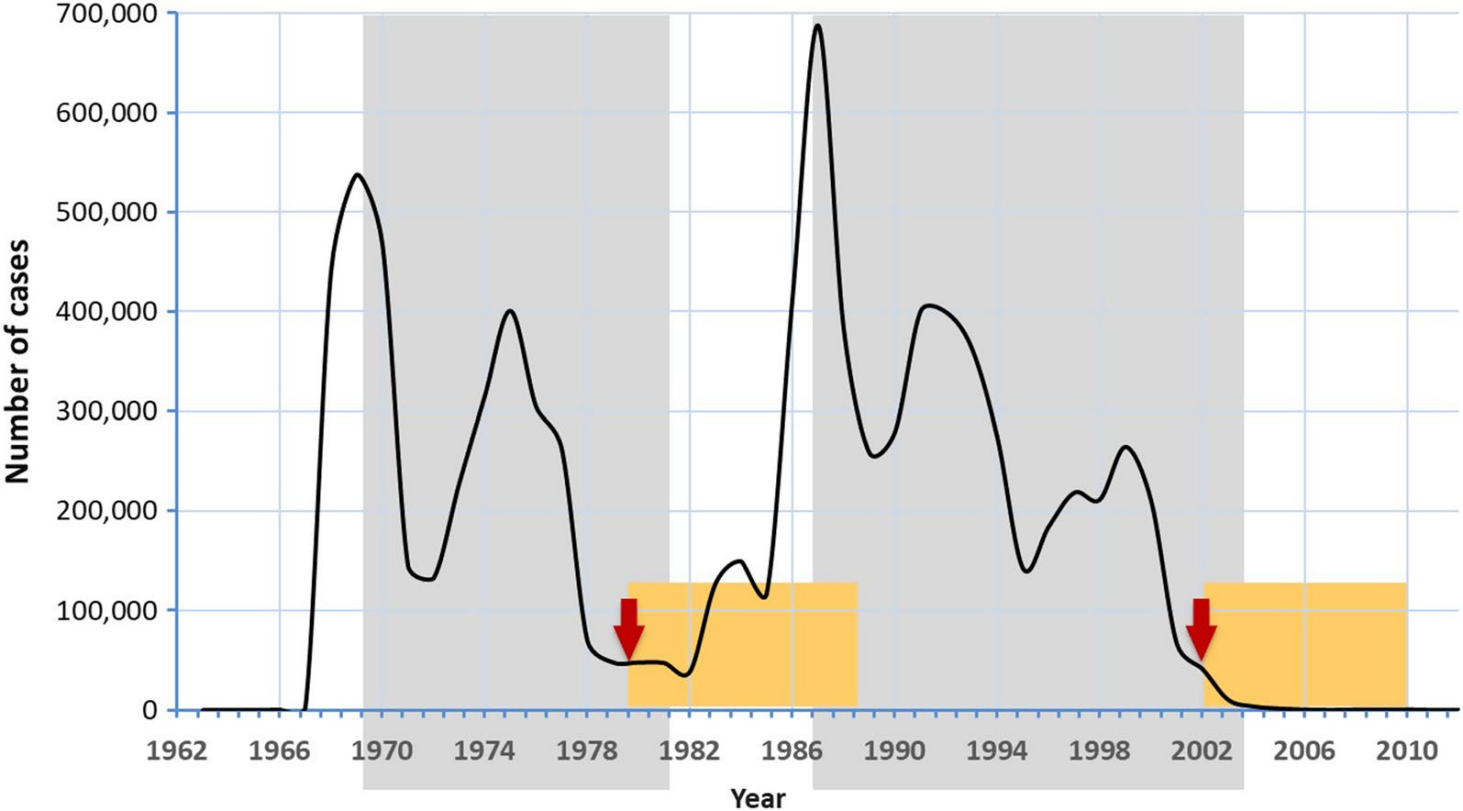
The annual malaria incidence in Sri Lanka 1962–2012 showing two very similar epidemic cycles in which the declining arms (area shaded in gray) were shown to be dynamically similar. Financial expenditure was compared in the 9 and 8 years (range of years shaded in yellow) which followed two pivotal years in this period (marked by arrows), in which the incidence was similar

The malaria incidence in Sri Lanka, as in the Southeast Asian region in general fluctuates seasonally as well as cyclically over periods of about 15–20 years (Fig. 1) depending on weather patterns, and possibly other unknown factors. This backdrop provides blocks of time with repeating sequences of malaria incidence patterns that lend themselves to comparison.

In this study, the financial costs incurred during the end of the last cycle in which an enhanced malaria control programme was in operation leading, eventually, to the interruption of transmission, was compared with a comparable period in a previous cycle of malaria in which a regular malaria control programme was underway but during which, contrastingly, the burden of malaria increased.

## Methods

### Strategic approach and data sources

Within the overall framework of a markedly fluctuating malaria incidence over the last 50 years there were two clear ‘epidemic’ cycles, 1967 to 1982 and 1982 to 2004 (Fig. 1). In order to ascertain if these two cycles had comparable transmission dynamics the rates of decline of incidence in the two cycles 1969 to 1982, and 1987 to 2004 were examined. The rates of change of incidence during the declining phases of these two cycles during the periods 1969–1982 (regression coefficient = − 1.7499) and 1987–2004 (regression coefficient = 0.175) were not significantly different (p = 0.096) demonstrating that the two cycles were indeed quite similar in terms of the dynamics of transmission.

Malaria incidence data and budgets, sources of funding and, expenditure data were obtained from the Anti Malaria Campaign, Ministry of Health, Sri Lanka from published reports [10, 11]. Expenditures in Sri Lankan rupees were converted to constant dollars of 2010 based on the exchange rate in a particular year and adjusting for the consumer price index as a proxy for inflation.

To compare the expenditure of the Anti Malaria Campaign in two different malaria situations, which followed the end of the two epidemics, periods of 8 to 9 years, one in each epidemic cycle, beginning each with a year in which the incidence was similar between the two cycles, and also relatively low, were chosen as shown in Fig. 1. These were the 9 years following 1980 the year in which the incidence was 47,961 and 8 years following 2002, the year in which the incidence was 41,411 cases per year (Fig. 1). The years 1980 and 2002 which marked the beginnings of these two periods were, in retrospect, decisive points in time because despite having a similar malaria incidence the disease took strikingly different courses in the years that followed; following 1980 the incidence rose rapidly to peak at nearly 700,000 cases whereas following 2002 the incidence decreased steadily up to the point of elimination.

## Results

**Fig. 2:**
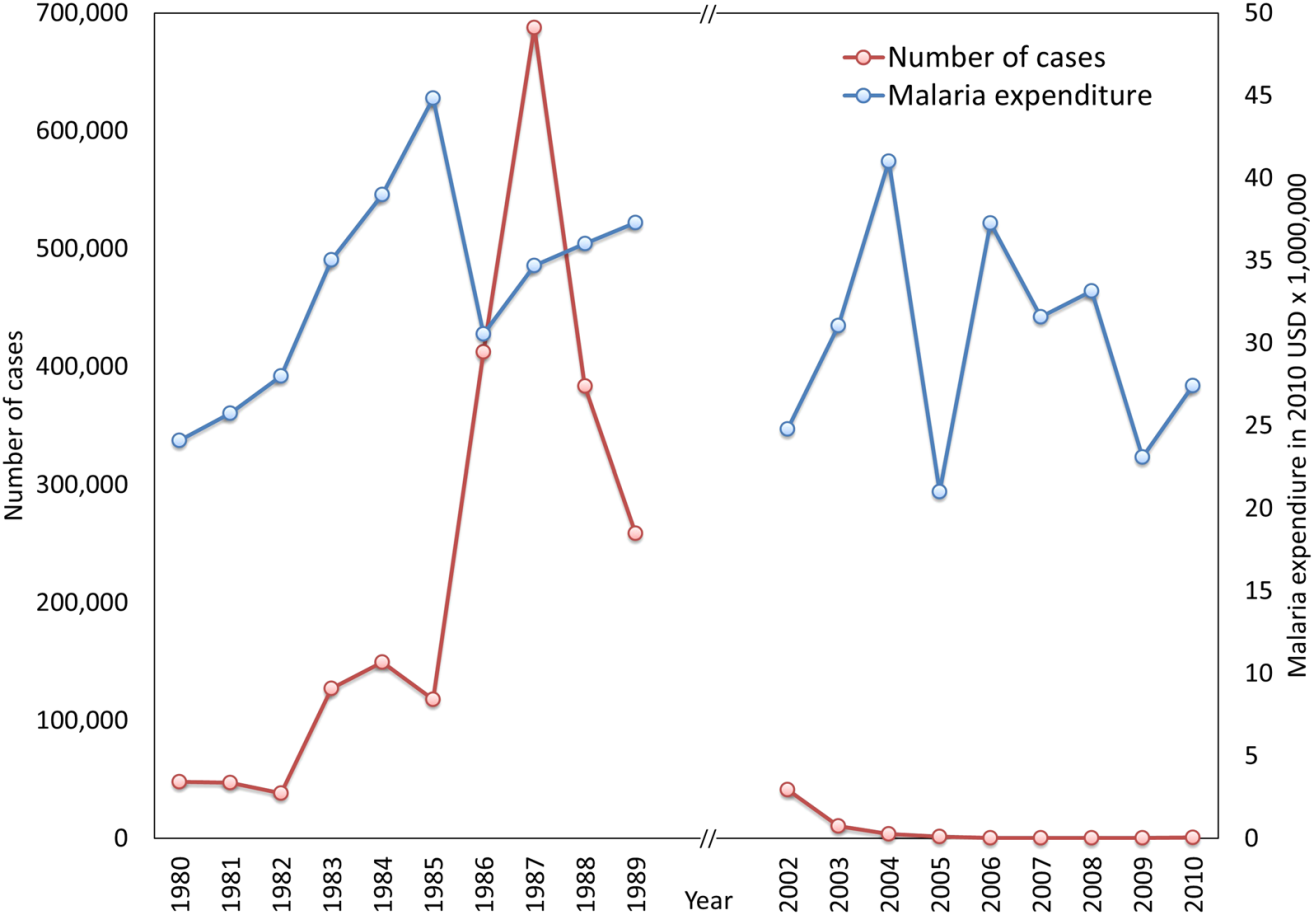
Malaria expenditure and the number of cases during the periods of malaria control (1980–1989) and malaria elimination (2002–2010)

**Fig. 3:**
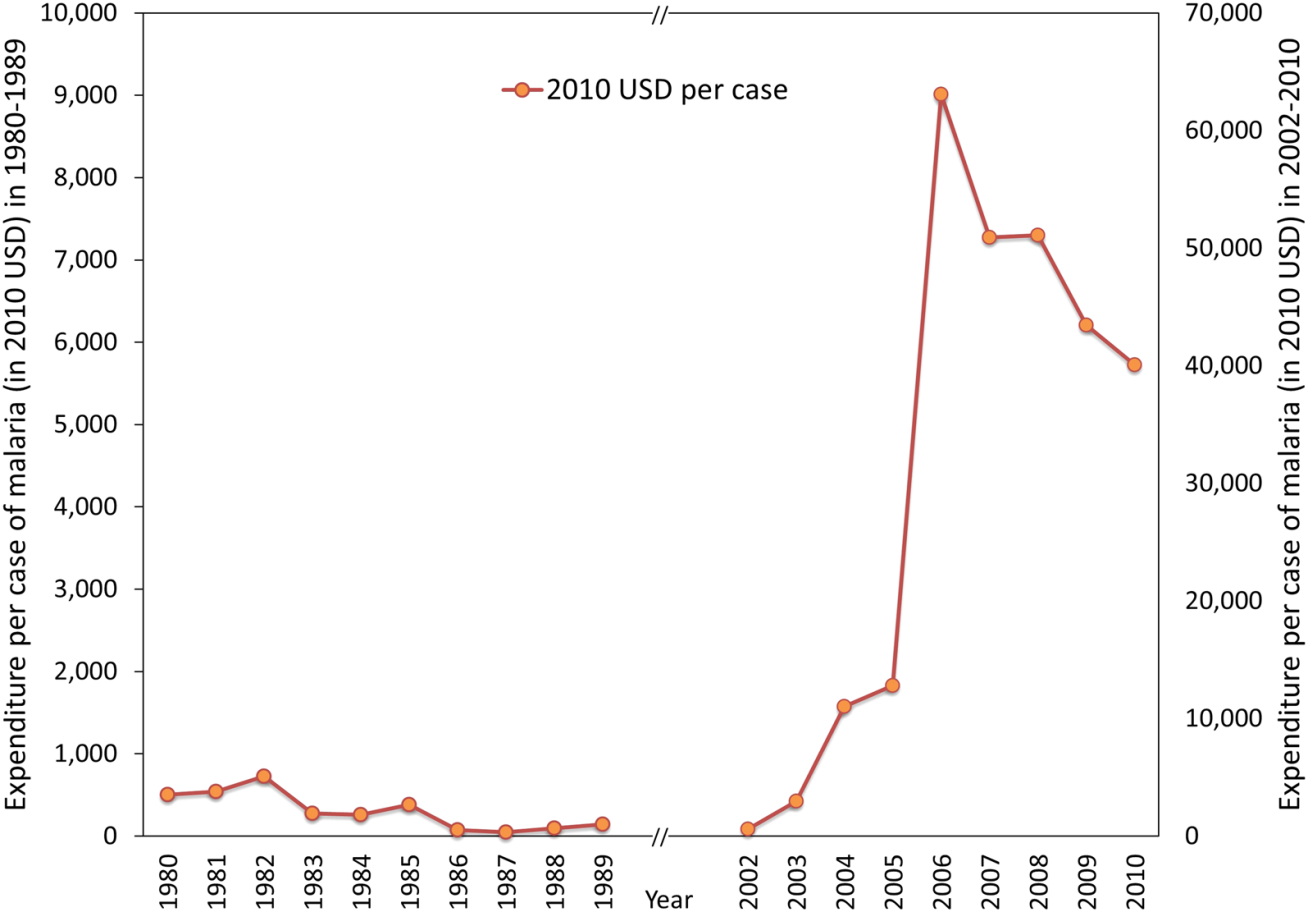
Expenditure per case of malaria in the two periods of malaria control (left) and elimination (right). Note that the vertical axes scales are different for the two time periods

The total expenditure of the malaria control programme was, with some yearly fluctuations, similar in both these periods being in the broad range of 21 to 45 million USD a year (adjusted to 2010 USD) (Mann Whitney U statistic = 31; z = 1.1023; p = 0.2713) (Fig. 2). Given that the malaria incidence during these two periods were quite dissimilar, the cost to the programme per case of malaria was correspondingly different in the two time periods (Fig. 3). In the 1980s, a single case of malaria cost the programme between 50 and 500 USD, and in the latter, it rose from a similar figure of 500 USD to the range of 40,000 to 52,000 USD per case.

**Fig. 4:**
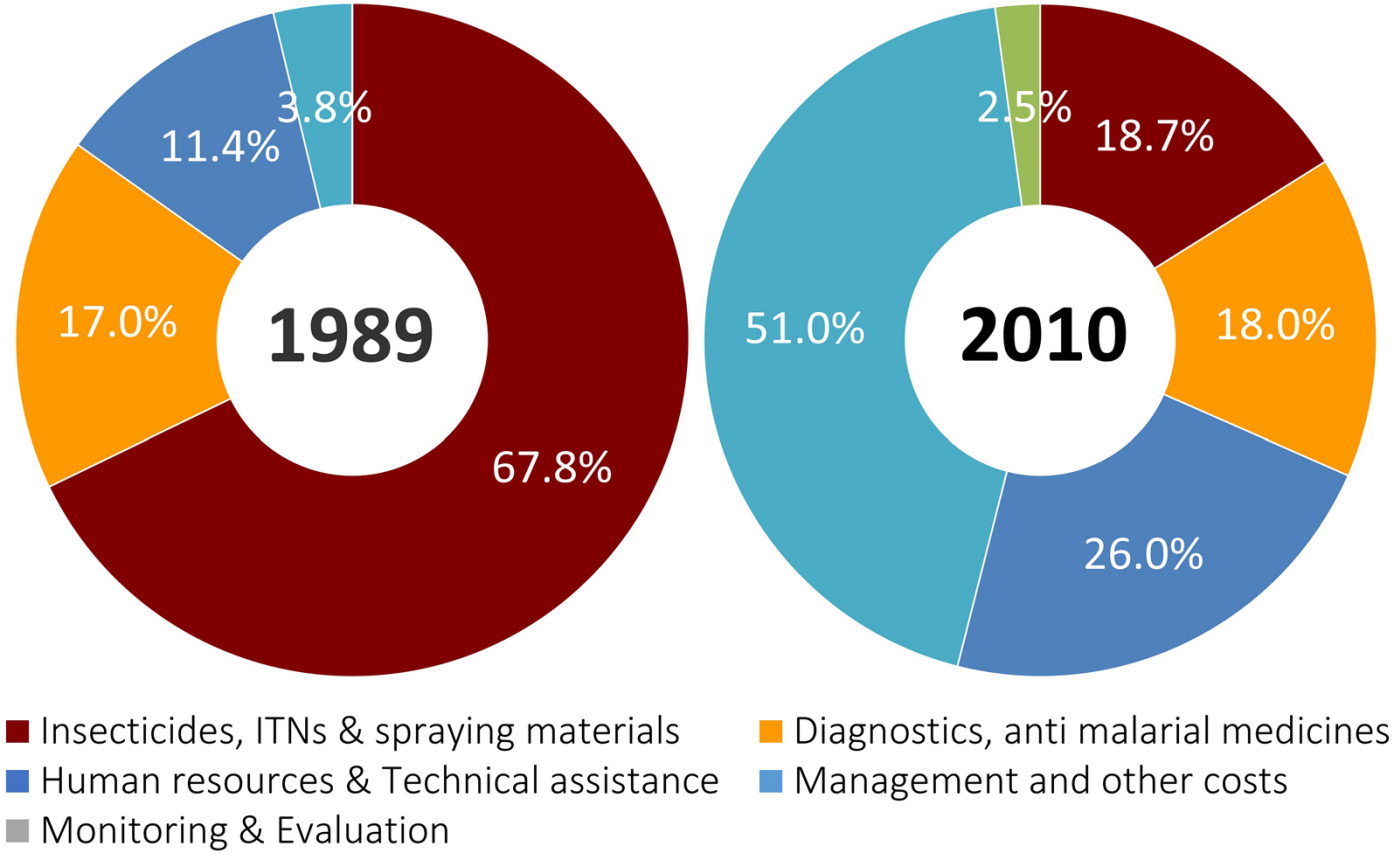
AMC expenditure (proportions) on budget categories in 1989 (during malaria control) and 2010 (during malaria elimination)

The financial sources that constitute the budget of the AMC are the national government and foreign funding which comprise international grants and bilateral aid from foreign governments. During both periods 1980s (control phase) and early 2000s (elimination phase) the component of foreign funding which supplemented national government funding of the malaria programme was proportionately similar (p = 0.548), and constituted, on average, 24% (range 13–38%) of the total budget of the AMC. However, the source of external funding was quite different in the two periods. In the 1980s (control phase), most of the external funds were from bilateral sources (United States Agency for International Development, Governments of the United Kingdom, Netherlands, and to a much lesser extent Japan, and multilateral funding for technical assistance from the WHO). In the 2000s (elimination phase), nearly all of external funding came as grants from the Global Fund to fight AIDS, Tuberculosis and Malaria (GFATM). The expenditure categories, i.e. commodities and services for which total funds of the AMC were expended also differed markedly in the two periods. Years 1989 and 2010 were taken at the end of the two periods as examples for comparison (Fig. 4). In 1989, the bulk of the total funding (85%) was spent on insecticides, diagnostic services and anti-malarial medicines whereas in 2010, most of the funds (77%) were expended on programme management, human resources and technical assistance; the proportion of the budget spent on insecticides (18.7%), and diagnostics and medicines (1.8%) in 2010 was quite small. It is noteworthy that in the 1980s (control phase) there was no budget line for monitoring and evaluation where as in the 2000s (elimination phase) 2.5% of funds were expended throughout the years on monitoring and evaluation.

The core structure of the AMC and its modus operandi remained largely unchanged from 1980 onwards through to the end of the elimination programme. The staff cadres of the AMC and their roles and responsibilities remained unchanged throughout this period.

## Discussion

In hindsight, 1980 and 2002 were pivotal years in the malaria history of Sri Lanka. They were both at the end of epidemic cycles, and both had low and very similar malaria incidence rates. Yet, 1980 was followed by a resurgence of malaria with case numbers peaking at nearly 700,000, 7 years later, and the other, 2002, was followed by a steady decline in malaria culminating in elimination 10 years later. Theoretically, and in terms of transmission dynamics, malaria elimination might have been equally feasible in the early 1980s, as it was in the 2000s, because the starting case incidences were very similar. Instead, an epidemic followed 1980 despite a regular malaria control programme being in operation, just as there was one in 2002. The reasons why malaria increased rather than decreased following 1980 and the contrary happened after 2002 are probably many, but key among them could have been the adequacy of financial investments in malaria control, and/or programme strategy and efficiency during those times, both of which are investigated here.

The launch by the WHO in 1998 of the Roll Back Malaria Initiative as a major partnership drive to reduce the global burden of malaria [12] led to a considerable increase in global investments for malaria control, in particular with the establishment of the GFATM in 2002 [13]. Sri Lanka received three grants for malaria control and elimination totaling USD 9.6 million during the period of 2003 to 2008. At the onset of this enquiry, it was assumed that increased funding be central to the achievement of malaria elimination in Sri Lanka. On the contrary, the total expenditure of the malaria control programme in the control phase during 1980–1989, which saw a dramatic increase in malaria, when adjusted for inflation, was not any different to the expenditure during the 2002–2010 period which pre-empted and spanned most of the malaria elimination effort (Fig. 2).

However, the sources of external funding, which constituted about a quarter of the total budget of AMC during both periods, were quite different in the two periods. In the 1980s, it was bilateral sources of external funding and in the 2000s it was grants from the GFATM.

Exploring what then, would have been different in the 2002–2010 period which led to elimination, it is reasonably certain that it was not changes in the structure of the AMC in terms of the staff cadres and their roles and responsibilities nor its *modus operandi*. It is difficult, if not impossible, to quantify programme efficiencies in the two periods with the data available and, therefore, the arguments presented below are based on qualitative information and the familiarity of the investigators with the malaria control programme over several past decades.

There were two key differences pertaining to programme implementation between the malaria elimination effort in the 2000s and the malaria control efforts in the 1980s. One was how the external funds were utilized. Although the quantum of funding was not different during the two periods, the bulk of the external funding which, during the elimination phase in the 2000s, came as Global Fund grants that allowed a degree of flexibility in its utilization, that national funds did not permit. For example, GF grant disbursement conditions allowed the recruitment of additional staff of any category and with any skills on a temporary basis as long as it was justifiable and within the framework of the national strategic plan. During this period, the AMC did change the staff profile considerably without affecting the permanent cadres. The budgetary rules linked to the GFATM grants also allowed for changes to be made in the expenditure profile of grant funds in accordance with the needs. During the control phase in the years between 1980 and 1989 when external funding was almost entirely from bilateral sources, funds were expended in accordance with government regulations which were rigid. The approved cadres of staff categories had to be adhered to, and bureaucracy made it difficult to make changes or supplement existing cadres or change course even if they were justified.

The second and probably the more important difference was that disbursement of GF funds was linked to a rigorous monitoring and evaluation framework on the basis of pre-defined indicators and a performance framework relating to progress—stringently measuring process, outputs and outcomes. It is likely that this was responsible for a considerable increase in programme efficiency in the elimination phase in the 2000s. Though in the 1980s reporting was an integral component of the programme, rigorous monitoring and evaluation was not. These differences are aptly reflected in the spending patterns of the two periods. The budget categories on which funds were expended during the two periods were strikingly different. During malaria control in the 1980s, insecticides, diagnostics and medicines consumed 85% of the total budget, and there was no expenditure on monitoring and evaluation. In the 2000s the spending shifted markedly to programme management, human resources and technical assistance (77%), and there was an explicit budget line for programme monitoring and evaluation, which in themselves reflect a major change in strategy on which operations were based.

Importantly, during the elimination phase spending on malaria was maintained despite the case numbers decreasing leading to a major increase in the cost per case. The cost per case during elimination being incredibly high can potentially undermine a malaria programme’s ability to secure consistent domestic funding when there are competing priorities, and if there is insufficient understanding of, or support for, a malaria elimination goal at higher levels of government. However, as this case illustrates, the funds were expended not on individual malaria cases unlike in the 1980s, but on programme management and human resources, which supported a state-of-the-art surveillance system, and on monitoring and evaluation of the programme. These are capacities that benefit the entire health system well beyond malaria, which further increases efficiencies. These are noteworthy aspects of a successful elimination programme.

Apart from flexibility in the use of funds, particularly for staff recruitment through the use of external funding, and increased programme efficiency impelled by the GFATM grant management system, the other most significant change in 2000s was probably the motivation for malaria elimination created by the heightened global interest in malaria. The enthusiasm created by WHO’s Roll Back Malaria Initiative in 1998 which aimed to reduce the burden of malaria, permeated down to country malaria control programmes. In contrast, the 1980s was a grim period globally for malaria control-countries had not recovered from the aftermath of the failed Global Malaria Eradication Programme (GMEP), and were also riddled with technical challenges of drug and insecticide resistance. A sense of helplessness had set-in in countries and the will to combat malaria, leave alone eliminate it, had dwindled.

Several other factors that may have accounted for the striking difference in outcomes from the efforts of the AMC-elimination versus epidemic, were considered. Improved technologies and products were available in the 2000s, which were not in the 1980s. Rapid diagnostic tests which enabled a malaria diagnosis to be made at the point-of-care were used in Sri Lanka from 2002 onwards, but microscopy-based diagnostic services were the most widely used method in the country’s robust health system and all malaria diagnoses were eventually confirmed by microscopy. Long-lasting insecticidal nets (LLINs) were used in Sri Lanka from 2004 onwards, although here again, there was as much use of Indoor Residual Spraying (IRS) which was the mainstay of vector control in the 1980s as there was dependency on LLINs, and coverage by both, were relatively low during the 2000s [8], because of the focal approach adopted for vector control at the time. Resistance of *Plasmodium falciparum* malaria to chloroquine was widespread globally in the 1980s and it was so in Sri Lanka as well, and this could have accounted for the increase in cases in the 1980s. However, *P. falciparum* accounted for less than 30% of malaria cases in Sri Lanka then, and the country was quick to switch to the alternative medicine sulfadoxine-pyrimethamine and so, it is unlikely to have been a major cause for the epidemic. Globally, highly effective artemisinin-based combination therapy (ACT) began to be deployed for malaria treatment in the early 2000s and it was as late as 2008 that Sri Lanka adopted an ACT, and then was only for the treatment of *P. falciparum* which accounted for a minority of infections [14]. It is surmised, therefore, that the unavailability of these products in the 1980s, though may have contributed to, is unlikely to have been a major factor in accounting, even collectively, for the difference in the outcomes of the AMC's efforts in the decade of the early 2000s which led to elimination and that of 1980 which led to an epidemic.

The stark implication of these findings is that, at least in the context of Southeast Asia and other regions with similar transmission dynamics, malaria elimination does not have to cost more, financially, than does malaria control. This finding provides a compelling case for eliminating malaria, over and above the considerable returns in terms of both economic and health benefits on an investment in eliminating malaria [15].

Most malaria endemic countries in Asia are targeting elimination in the near or mid-term future. The Sri Lankan experience shows, that provided there is adequate and sustained funding, what is critical to achieve malaria elimination under these transmission conditions may be a responsive and agile programme which is ready and able to change course and technical strategy in response to a changing malaria situation, for which the financial support, or at least a component of it, needs to be flexible in its disbursement. Malaria elimination will also require vast improvements in programme management and efficiency. Mechanisms to support these changes were inherent in the Global Fund’s funding model. In the 1980s malaria control in Sri Lanka, apart from having as its goal, reducing malaria to a low level rather than elimination, had neither flexibility to be responsive to the malaria situation, nor the strategic approach required to bend the malaria curve, and malaria rose again subjected to environmental forces, despite the investment of an equivalent amount of funds.

It is well known that poor programme management resulting in inefficiencies are rife within public sector disease control programmes, particularly in developing countries. Improved management of disease control progammes with monitoring and evaluation, and flexibility of fund utilization are solely needed to get more returns from financial investments in public health. Unfortunately, these softer attributes of public health programmes are too often ignored in favour of tools, technical strategies and financial investments, which are more tangible and easier to count, measure and report on. The findings of this study also suggest that donor funds for health programmes may yield better outcomes if they are channeled through well-managed multilateral global health funding agencies such as the GFATM, than through bilateral avenues.

## Conclusion

Malaria elimination in Southeast Asia may not require more funding than malaria control. However, an agile programme with flexibility in fund utilization and improved efficiencies in programme management with stringent monitoring and evaluation are critical requirements.

## Data Availability

All data used are published secondary data which have been referenced. All the data have been indicated in the figures.

## Abbreviations

AMC: Anti-malaria campaign, Sri Lanka
ACT: Artemisinin-based combination therapy
GF: Global Fund
GFATM: Global Fund to fight AIDS, Tuberculosis and Malaria
GMEP: Global Malaria Eradication Programme
IRS: Indoor Residual Spraying
LLIN: Long-lasting insecticidal nets
USD: United States dollars
WHO: World Health Organization
